# PTTG1 Attenuates Drug-Induced Cellular Senescence

**DOI:** 10.1371/journal.pone.0023754

**Published:** 2011-08-17

**Authors:** Yunguang Tong, Weijiang Zhao, Cuiqi Zhou, Kolja Wawrowsky, Shlomo Melmed

**Affiliations:** Department of Medicine, Cedars-Sinai Medical Center, University of California Los Angeles, Los Angeles, California, United States of America; University of Nebraska Medical Center, United States of America

## Abstract

As PTTG1 (pituitary tumor transforming gene) abundance correlates with adverse outcomes in cancer treatment, we determined mechanisms underlying this observation by assessing the role of PTTG1 in regulating cell response to anti-neoplastic drugs. HCT116 cells devoid of PTTG1 (PTTG1^−/−^) exhibited enhanced drug sensitivity as assessed by measuring BrdU incorporation *in vitro*. Apoptosis, mitosis catastrophe or DNA damage were not detected, but features of senescence were observed using low doses of doxorubicin and TSA. The number of drug-induced PTTG1^−/−^ senescent cells increased ∼4 fold as compared to WT PTTG1-replete cells (p<0.001). p21, an important regulator of cell senescence, was induced ∼3 fold in HCT116 PTTG1^−/−^ cells upon doxorubicin or Trichostatin A treatment. Binding of Sp1, p53 and p300 to the p21 promoter was enhanced in PTTG1^−/−^ cells after treatment, suggesting transcriptional regulation of p21. p21 knock down abrogated the observed senescent effects of these drugs, indicating that PTTG1 likely suppresses p21 to regulate drug-induced senescence. PTTG1 also regulated SW620 colon cancer cells response to doxorubicin and TSA mediated by p21. Subcutaneously xenografted PTTG1^−/−^ HCT116 cells developed smaller tumors and exhibited enhanced responses to doxorubicin. PTTG1^−/−^ tumor tissue derived from excised tumors exhibited increased doxorubicin-induced senescence. As senescence is a determinant of cell responses to anti-neoplastic treatments, these findings suggest PTTG1 as a tumor cell marker to predict anti-neoplastic treatment outcomes.

## Introduction

PTTG1 (pituitary tumor transforming gene), originally isolated from rat pituitary tumor cells [1], is a securin protein abundantly expressed in hematopoietic, colorectal, thyroid, lung, breast and ovarian malignancies [2]. PTTG1 was proposed as a marker of breast cancer aggressiveness [3], [4], [5]. Increased PTTG1 expression correlates with higher colorectal tumor grade, invasion of surrounding lymph nodes and increased tumor vascularity [6], [7].

PTTG1 is a natively unfolded protein [8] which binds to ∼700 gene promoters as indicated by ChIP-on-Chip assay [9], and interacts with ∼80 proteins [10]. In addition to its securin function [11], PTTG1 regulates G2/M phase transition by inhibiting separase cleavage and aurora kinase A activity [10], [12]. PTTG1 also regulates G1/S transition by inducing cyclin D3 and repressing p21 expression [9]. PTTG1 induces FGF2, stimulates c-myc expression, inhibits p53 DNA binding and transactivation and regulates apoptosis [13], [14], [15], [16], [17]. PTTG1 over-expression leads to cell transformation in NIH-3T3 cells, while mouse embryo fibroblast cells devoid of *Pttg* are resistant to oncogenic transformation [9]. Targeted pituitary PTTG over-expression in *Rb^+/−^* mice resulted in enhanced cellular activity, increased gland volume, and higher prevalence of anterior pituitary tumors [18], [19]. Conversely, transgenic *pttg* deletion abrogated murine pituitary tumor development [20].

Recent research indicated that PTTG1 regulates cancer cell growth responses to treatments. Head and neck squamous cell carcinomas with higher tumor PTTG1 levels exhibit increased recurrences after radiation, 5-fluorouracil (5-FU) and cisplatinum treatment [21]. Breast cancers with higher PTTG1 levels are resistant to tamoxifen and fulvestrant [22]. Reports on gliomas [23], hepatocellular [24], thyroid [25] and esophageal carcinoma patients [26] also indicate that higher tumor PTTG1 levels correlate with adverse clinical outcomes.

PTTG1 has been implicated in mediating oncogene-induced pituitary tumor senescence [27]. As senescence also determines cancer therapy outcomes, we examined whether PTTG1 regulates drug-induced cellular senescence. Here we show here that the presence of PTTG1 is a determinant of drug-induced senescence. These findings contribute to understanding the requirement of PTTG1 for regulation of cancer cell response to anti-neoplastic drugs.

## Materials and Methods

### Cell lines and reagents

HCT116 is a human colon carcinoma cell line, expressing wild-type p53 and PTTG1 genes, and HCT116 p53^−/−^ and HCT116 PTTG1^−/−^ cell lines were derived using homologous recombination to knock out p53 or PTTG1 respectively [28], [29]. Cells were cultured in McCoy's 5A medium containing 15% FBS. SW620 human colon cancer cells were purchased from ATCC (Manassas, VA) and cultured as recommended. PTTG1 and p300 siRNA were purchased from Ambion (Austin, TX), p21 and p53 siRNA from Cell Signaling (Danvers, MA), PTTG antibody from Invitrogen (Carlsbad, CA), p300 and HDAC1 antibodies from Santa Cruz (Santa Cruz, CA), p21 antibody from Cell Signaling, and p53 antibody from CalBioChem (Gibbstown, NJ).

### BrdU incorporation assay

The BrdU incorporation assay was carried out using Cell Proliferation ELISA kits from Roche (Indianapolis, IN). Briefly, 2000 HCT116 cells or 3000 SW620 cells were cultured in 96-well microplates in a final volume of 100 ul culture medium per well, drugs added and cells incubated for 48 hours. BrdU labeling solution was added to a final concentration of 10 uM, and cells incubated for an additional 4 hours at 37°C. The culture medium was removed and each well washed twice with 250 ul wash medium containing 10% serum. Cells were fixed and treated with nucleases working solution for 30 min at 37°C. Incorporated BrdU was detected using anti-BrdU-POD, Fab fragments and peroxidase substrate ABTS. Sample absorbance was measured at 405 nm against background control, using a Victor 3 multiwell plate reader. Inhibition rate was calculated for each well as (A405 control cells - A405 treated cells)/A405 control cells×100% (A405: OD value at 405 nm)

### Senescence detection assay

SA-β-gal enzymatic activity was detected using a Senescence Cell Staining kit from Sigma-Aldrich (St. Louis, MO). Briefly, 10,000 cells were plated to 12-well plate, incubated at 37°C overnight and treated with control vehicle or drugs. Forty eight hours after treatment, cells were washed with PBS (pH 6.0), fixed overnight, and stained with 5-bromo-4- chloro-3-indolyl-h-D-galactopyranoside (X-Gal) overnight at 37°C. Ten fields were randomly selected and cells counted. Senescent rate was calculated for each field as positive cells/all cells×100%.

### Tunel assay

Tunel assay was carried out using in situ cell death detection kit from Roche. Briefly, 10,000 cells were plated to 12-well plate, incubated at 37°C overnight and treated with control vehicle or drugs. Forty eight or 96 hours after treatment, control and drug-treated cells were trypsinized and resuspended in 100 ul fixation solution for 30 minutes Cells were permeabilized, washed and incubated in TUNEL assay mixture at 37°C for 1 hour. Cells were washed, incubated in 10 ul 1× PBS+0.5 mg/ml propidium iodide (PI) for 10 min and subject to microscopic analysis. Ten fields were randomly selected and cells counted. Apoptotic rate was calculated for each field as positive cells/all cells×100%.

### Western blot

Protein extracts were resolved by Nupage 4–12% Bis-Tris Gel (Invitrogen), samples electro-blotted onto PVDF membrane (Invitrogen), and membranes blocked and incubated with primary antibody. Donkey anti-rabbit or anti-mouse (GE Healthcare, NJ) antibodies were conjugated to horseradish peroxide to reveal immunocomplexes by enhanced chemiluminescence (Pierce, IL). The detected bands were quantified using Image J v1.43 as instructed in the software manual.

### ChIP assay

Chromatin immunoprecipitaton was performed with ChIP-IT kits (Active Motif, Carlsbad, CA). Briefly, cells were fixed and chromatin sonicated to 0.3–2 kb with most at 0.8 kb. A negative IgG provided in the kit was used as a negative control antibody. Chromatin samples were immunoprecipitated using depicted antibodies, RNA removed by addition of Rnase A and cross-links removed by incubating at 65°C for 4 hours. Proteins were removed by treating with proteinase K, and DNA purified and subjected to realtime PCR detection.

### Luciferase assay

p21 promoter activity assay was carried out using Dual Luciferase Reporter System (Promega) according to the manufacturer's protocol. Briefly, ten thousand cells were seeded in 24-well plates and incubated at 37°C overnight. Each well was co-transfected with 200 ng luciferase plasmids (pGL3-Basic as control, p21 promoter with/out p53 binding sites) and 5 ng pRL-TK plasmids (Promega) encoding Renilla (internal control to assess transfection efficiency). After 24 hours, whole-cell lysates were collected and measured using an Orion Microplate Luminometer (Berthold Detection System). Transfections were performed in triplicate and repeated three times to assure reproducibility. All luciferase reads were normalized to corresponding Renilla value. Relative luciferase activity was calculated using the following formula: Relative luciferase levels = Luc-promoter/Luc-pGL3 basic.

### Subcutaneously xenografted tumor

Animal protocols were approved by the Cedars Sinai Institutional Animal Care and Use Committee (protocol # 2603). 5- to 6-week-old female athymic mice were separated into two groups, one for HCT116 WT and another for HCT116 PTTG1^−/−^ cells. Cells (0.1 ml Matrigel containing 1×10^6^ cells) were injected subcutaneously into the lumbar area at left side to generate tumors. Tumors were allowed to grow when the first tumor reaching ∼100 mm^3^, at which time mice were further randomized to 2 treatment groups, with 10 evaluable tumors in each group: (a) control and (b) drug treated. Doxorubicin was administered iv through the tail vein (10 mg/kg) at day 1 after grouping. Mice were monitored daily for signs of toxicity and weighed twice weekly. Tumor sizes were measured twice weekly by caliper measurements using the following formula: tumor volume =  (length×width^2^) /2 as previously reported [30], [31]. Relative tumor growth was calculated by relative tumor size of treated mice divided by relative tumor size of control mice (T/C). At the end of the study, 3 weeks after cell injection, animals were euthanized under CO_2_ inhalation followed by cervical dislocation to ensure death (consistent with the Panel on Euthanasia of the American Veterinary Medical Association). Tumor tissue was collected, weighed and cut into three pieces for analysis. One was used for mRNA extraction, one was frozen immediately in liquid nitrogen for protein analysis and the third one fixed with formaldehyde, embedded in paraffin and sectioned.

### Immunofluorescence

Tissue sections were deparaffinized in xylene, hydrated in graded ethanol, and heated in Target Retrieval Solution (DakoCytomation) to retrieve antigen at 95°C for 40 minutes. Slides were permeabilized with 1% triton-X-100 in PBS for 30 minutes, and incubated in blocking buffer (10% goat serum, 1% BSA, 0.1% triton-X-100 in PBS) for 1 hour. After washing with PBS, slides were hybridized at 4°C overnight with antibodies against indicated proteins. Primary antibody was omitted for negative controls. Alexa Fluor anti-mouse antibody with green fluorescence or anti-rabbit antibody with red fluorescence (Molecular Probes, Carlsbad, CA) were used as secondary antibodies and incubated at room temperature avoiding light for 1 hour. Slides were mounted with ProLong Gold Antifade Reagent with DAPI (Invitrogen), and nuclei dyed by DAPI with blue fluorescence. Senescence activity in tissue samples was assessed by measuring p21 expression and β-gal activity. Ten fields were randomly selected and cells counted. Senescent or apoptotic rate was calculated for each field as positive cells/all cells×100%.

### Data analysis

All results were expressed as mean±SD. Two group comparison was assessed by non-paired, two tail Student's t-test. The difference between multiple groups was assessed by ANOVA.

## Results

### HCT116 PTTG1^−/−^ cells exhibit enhanced drug-induced senescence

**Figure 1:**
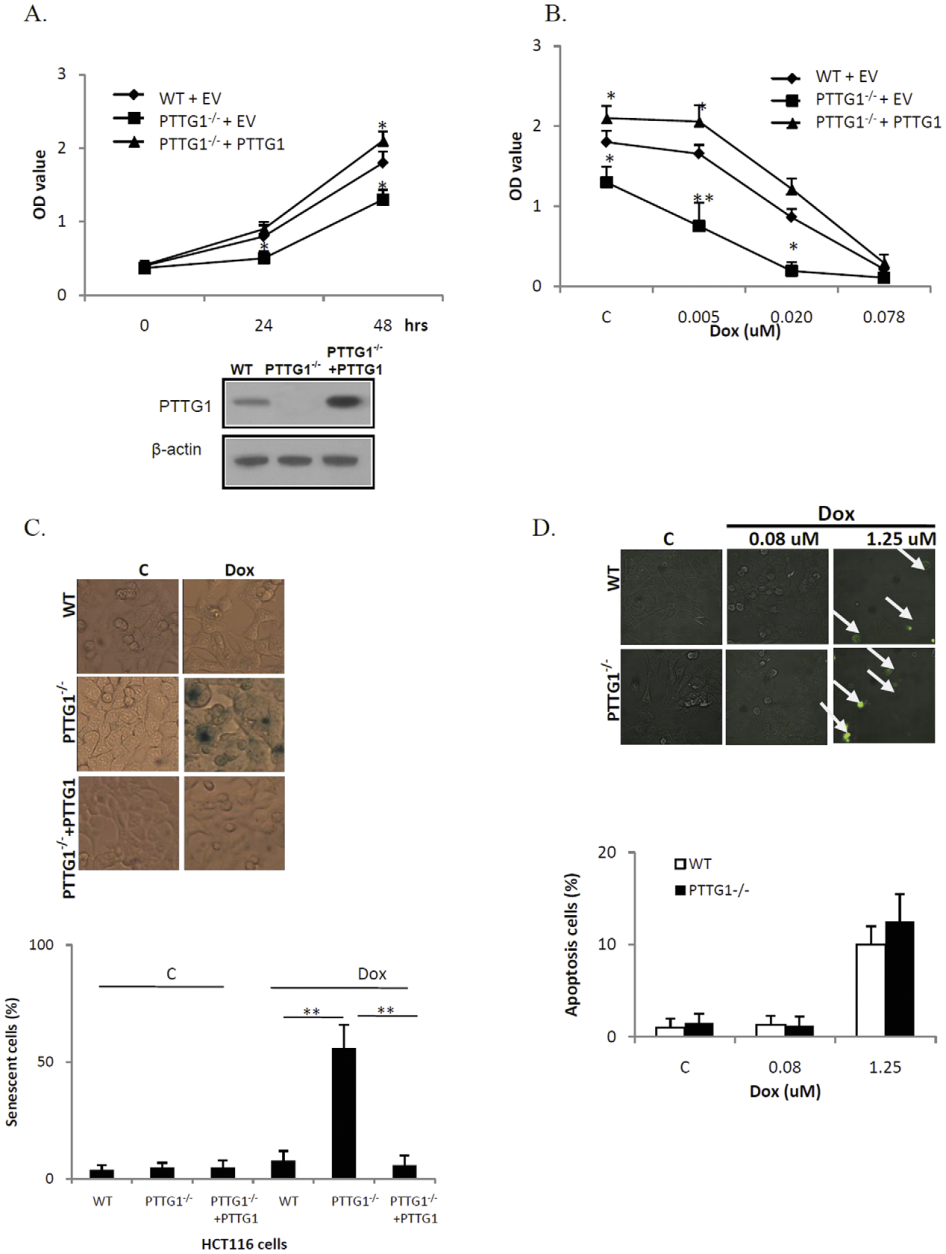
HCT116 PTTG1^−/−^ cells are more sensitive to doxorubicin (Dox). a) HCT116 WT and PTTG1^−/−^ cells expressing an empty vector (WT+EV and PTTG1^−/−^ +EV), and PTTG1 re-introduced PTTG1^−/−^ (PTTG1^−/−^ +PTTG1) cells were plated in 96-well plates and DNA synthesis assessed at different time points (time 0 represents 0 hours after cell plating); b) HCT116 WT and PTTG1^−/−^ cells expressing an empty vector (WT+EV and PTTG1^−/−^ +EV), and PTTG1 re-introduced PTTG1^−/−^ (PTTG1^−/−^ +PTTG1) cells were plated in 96-well plates for 24 hours and treated with control vehicle (C) or different doxorubicin doses for another 48 hours. DNA synthesis was assessed by measuring BrdU incorporation; c) HCT116 WT and PTTG1^−/−^ cells were plated for 24 hours and treated with control vehicle (C) or doxorubicin (0.02 uM) for another 48 hours, fixed, stained for β-gal activity and cells counted; d) HCT116 WT and PTTG1^−/−^ cells were plated for 24 hours and treated with control vehicle (C) or different doses of doxorubicin for another 48 hours, Tunel assay performed and positive cells counted. Each experiment was repeated three times. Results expressed as mean±SD, n = 3, *p<0.05, **p<0.001.

We evaluated WT HCT116 and PTTG1^−/−^ cell proliferation by measuring BrdU incorporation. As shown in Fig. 1a, with similar cell numbers plated, HCT116 WT and PTTG1^−/−^ cells exhibited similar absorbance (OD 0.4) at 0 hour. After 48 hours, HCT116 WT cells showed an absorbance of 1.8 and PTTG1^−/−^ cells 1.3 (28% decrease compared to WT, figure S1), suggesting that HCT116 PTTG1^−/−^ cell number were less than WT. Reintroduction of PTTG1 into HCT116 PTTG1^−/−^ cells (PTTG1^−/−^ +PTTG1) increased cell number after 48 hours (see figure S2 for transfection efficiency), and these cells exhibited an OD value of 2.1 (1.6 fold increase compared to PTTG1^−/−^ expressing an empty vector). We then evaluated doxorubicin-treated HCT116 WT and PTTG1^−/−^ cells. As shown in Fig. 1b, doxorubicin (0.005 uM) inhibited BrdU incorporation by 8% in WT cells (OD value reduced from 1.8 in control vehicle group to 1.7 in doxorubicin group, not significant), and 42% in PTTG1^−/−^ cells (OD value reduced from 1.3 in control vehicle group to 0.8 in doxorubicin group, p<0.001). Treatment with higher doses (0.02 uM) of doxorubicin inhibited BrdU incorporation by 52% in WT cells and 85% in PTTG1^−/−^ cells (p<0.05). As lower doses (<0.1 uM) of doxorubicin cause senescence [32], and HCT116 PTTG1^−/−^ cells were more sensitive to low dose doxorubicin as measured by BrdU incorporation, we reasoned that HCT116 PTTG1^−/−^ cells may undergo enhanced drug-induced senescence. We therefore performed β-galactosidase (β–Gal) staining as a marker to evaluate cell senescence. As indicated in Fig. 1c, few cells stained positively when treated with control vehicle. After doxorubicin (0.005 uM) treatment, 56% of HCT116 PTTG1^−/−^ cells exhibited enhanced β–Gal staining, compared to 5% of WT cells. We then tested whether apoptosis was involved in regulating HCT116 PTTG1^−/−^cells response to doxorubicin. HCT116 WT and PTTG1^−/−^ cells were Tunel assay positive only when treated with higher doxorubicin doses (1.25 uM, Fig. 1d). Cleaved caspase 3 was not detected in doxorubicin (0.005–0.02 uM) treated HCT116 WT cells even after 96 hours treatment (data not shown). Thus enhanced PTTG1^−/−^ cell sensitivity to low doxorubicin doses is likely due to enhanced senescence rather than apoptosis. We detected ∼51% increase of doxorubicin-induced senescence, but only ∼30% decrease in cell numbers when treated with 0.02 uM doxorubicin. This result might be due to the fact that senescent cells are still metabolically viable.

### Over-expressing PTTG1 attenuates drug-induced senescence

To assess whether enhanced senescence and cell sensitivity could be rescued, we reintroduced PTTG1 into HCT116 PTTG1^−/−^ cells, and observed modest inhibition of BrdU incorporation as compared to HCT116 PTTG1^−/−^ cells (42%, OD value reduced from 1.3 to 0.75) when treated with 0.005 uM doxorubicin (p<0.001) (Fig. 1b). β–Gal staining indicated fewer senescent cells when PTTG1 was re-introduced (6%) than in control HCT116 PTTG1^−/−^ cells (56%, p<0.001, Fig. 1c).

**Figure 2:**
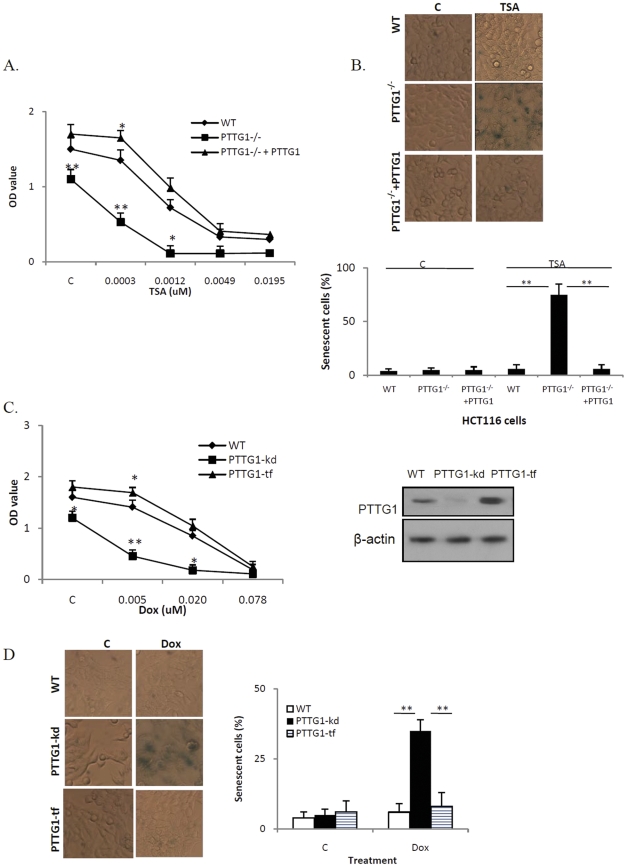
HCT116 PTTG1^−/−^ and SW620 PTTG1 knock down (PTTG1-kd) cells exhibit enhanced drug-induced senescence. a) HCT116 WT, PTTG1^−/−^ and PTTG1 re-introduced PTTG1^−/−^ (PTTG1^−/−^ +PTTG1) cells were plated in 96-well plates for 24 hours and treated with control vehicle (C) or TSA for another 48 hours. DNA synthesis was assessed by measuring BrdU incorporation; b) HCT116 WT and PTTG1^−/−^ cells were plated for 24 hours and treated with control vehicle (C) or TSA (0.005 uM) for another 48 hours, fixed, stained for β-gal activity and cell number counted; c) SW620 WT, PTTG1 knock down (PTTG1-kd) cells and PTTG1-transfected (PTTG1-tf) cells were plated in 96-well plates for 24 hours and treated with control vehicle (C) or doxorubicin (Dox) for another 48 hours, DNA synthesis was assessed by measuring BrdU incorporation; d) SW620 WT, PTTG1 knock down (PTTG1-kd) cells and PTTG1 transfected (PTTG1-tf) cells were plated in 24-well plates for 24 hours, treated with control vehicle (C) or doxorubicin (0.02 uM) for another 48 hours, β-gal staining performed and positive cells counted. Each experiment was repeated three times. Results are expressed as mean±SD, n = 3, *p<0.05, **p<0.001.

Trichostatin A (TSA) selectively inhibits histone deacetylase (HDAC) enzymes [33], interfering with removal of histone acetyl groups thereby altering transcription factor access to DNA. As TSA induces senescence in several cell lines without causing DNA damage [34], [35], we tested whether HCT116 PTTG1^−/−^ cells are more sensitive to TSA. As shown in Fig. 2a, treatment with 0.3 nM TSA inhibited BrdU incorporation by 52% (OD value reduced from 1.1 to 0.53) in HCT116 PTTG1^−/−^ cells and 10% (OD value reduced from 1.5 to 1.35) in WT cells (p<0.001). β-gal staining showed that 75% of treated HCT116 PTTG1^−/−^ cells were senescent compared to 5% of treated WT cells (p<0.001, Fig. 2b). When PTTG1 was reintroduced to PTTG1^−/−^ cells, the BrdU incorporation inhibition by TSA was decreased to 6% (p<0.001 compared to 52% in PTTG1^−/−^ cells, Fig. 2a). The induction of senescence was decreased to 6% (p<0.001 compared to 75% in PTTG1^−/−^ cells, Fig. 2b), suggesting that PTTG1 attenuates the cell response to TSA treatment.

We then tested whether PTTG1 attenuates drug responsiveness and drug-induced senescence in other cell types. SW620 colon cancer cells transfected with empty vector or PTTG1 plasmids or PTTG1 siRNA were treated with doxorubicin. In control vehicle treated cells, PTTG1 knock down cells (PTTG1-knock down) exhibited lower OD value (1.2) than WT (1.6, p<0.05 vs PTTG1-knock down) and PTTG1 transfected cells (1.6, p<0.05 vs PTTG1-knock down), suggesting that knock down of PTTG1 inhibited SW620 cell proliferation (Fig. 2c). After treatment with doxorubicin (0.005 uM), BrdU incorporation was inhibited by 62% (OD value reduced from 1.2 to 0.46, p<0.001), compared to 12% (OD value was reduced from 1.6 to 1.4) in WT cells. Senescence was enhanced in PTTG1-knock down cells (35%, p<0.05, Fig. 2d). SW620 cells transfected with PTTG1 exhibited inhibition of BrdU incorporation of 6% (OD value reduced from 1.8 to 1.69). In control (control vehicle-treated) group, few cells appeared senescent. These results indicate that PTTG1 attenuates SW620 colon cancer cell senescence and responsiveness to doxorubicin.

### p21 and drug-induced HCT116 PTTG1^−/−^ cell senescence

**Figure 3:**
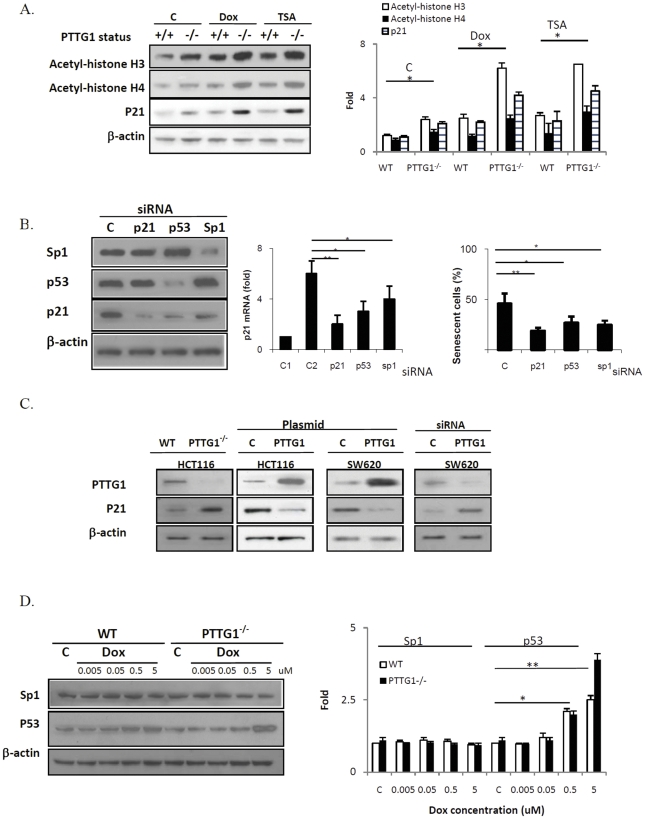
PTTG1 down regulates p21 in colon cancer cells. a) HCT116 WT and PTTG1^−/−^ cells were treated with control vehicle (C), doxorubicin (Dox, 0.02 uM) or TSA (0.005 uM), and proteins analyzed by Western blotting to assess histone H3 and H4 acetylation; b) PTTG1^−/−^ cells were transfected with scrambled siRNA (C), or siRNA targeting p21, p53 or Sp1 respectively and treated with doxorubicin (0.02 uM) for 48 hours. Protein levels were evaluated by Western blotting and senescence evaluated with β-gal staining; c) HCT116 or SW620 cells were transfected with empty vector or scrambled siRNA (C) or PTTG1 plasmids or siRNA as indicated, proteins were extracted and PTTG1 and p21 levels evaluated by Western blotting; d) WT and PTTG1^−/−^ cells were treated with control vehicle (C) or different doses of doxorubicin (0.005, 0.05, 0.5 and 5 uM) for 48 hours Sp1 and p53 levels analyzed by Western blotting. Each experiment was repeated three times. Results are expressed as mean±SD, n = 3, *p<0.05, **p<0.001.

Doxorubicin-induced senescence is likely due to histone acetylation induced p21 elevation rather than DNA damage [35]. We evaluated the relative contribution of these two mechanisms to our observations. Consistent with previous reports [35], cells treated with low dose doxorubicin or TSA did not exhibit H2AX phosphorylation (data not shown), suggesting that DNA damage is not directly involved in drug-induced senescence. Chromatin acetylation facilitates access of transcriptional machinery to gene promoter regions [36], and we observed higher basal and treatment-induced acetylated histone H3 and H4 levels measured by western blot in HCT116 PTTG1^−/−^ cells (Fig. 3a). To test the requirement of p21 for enhanced senescence observed in PTTG1^−/−^ cells, we knocked down p21 and then assessed effects of cell treatments. PTTG1^−/−^ HCT116 cells exhibited 21% senescent cells when p21 was suppressed, compared to 58% in control siRNA treated cells (p<0.001) (Fig 3 b), indicating the requirement for p21 in PTTG1 mediated senescence. Moreover, HCT116 PTTG1^−/−^ cells possess higher p21 levels. Over expressing PTTG1 reduces p21 levels in both HCT116 and SW620 cells (Fig 3 c).

### Sp1 and p53 regulate p21 expression and senescence in PTTG1^−/−^ cells

**Figure 4:**
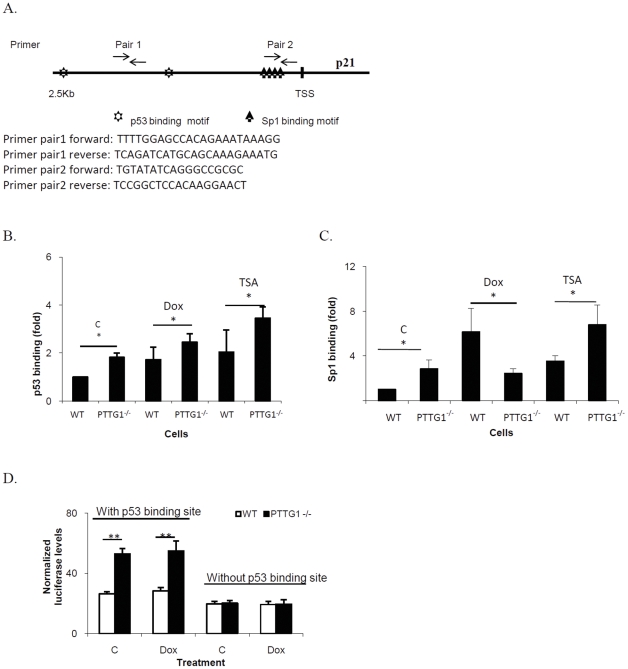
Sp1 and p53 regulate p21 levels and promoter activities in HCT116 WT and PTTG1^−/−^ cells. a) Primer pair 1 was designed to detect protein binding close to p53 motifs on p21 promoter. Primer pair 2 was used to detect protein binding close to Sp1 binding site on p21 promoter; b) WT and PTTG1^−/−^ cells treated with control vehicle (C), doxorubicin (Dox, 0.02 uM) or TSA (0.005 uM) for 48 hours and chromatin immunoprecipitation performed using p53 antibody. Enriched chromatin was analyzed by qPCR using primer pair 1; c) WT and PTTG1^−/−^ cells were treated with control vehicle (C), Dox (0.02 uM) or TSA (0.005 uM) for 48 hours and chromatin immunoprecipitation performed using Sp1 antibody. Enriched chromatins were analyzed by qPCR using primer pair 2; d) luciferase plasmids containing p21 promoters (with/out p53 binding motifs) were transfected into drug-treated HCT116 WT and PTTG1^−/−^ cells. Cells were harvested and luciferase activity analyzed. Each experiment was repeated three times. Results are expressed as mean±SD, n = 3, *p<0.05, **p<0.001.

Sp1 and p53 regulate p21 transcription [37], while PTTG1 enhances in vitro Sp1 binding [9] and also inhibits p53 transactivating activity [16]. We therefore evaluated p53 and Sp1 protein levels and show that HCT116 PTTG1^−/−^ cells contain similar levels of p53 and Sp1 compared to WT cells (Fig. 3d). After treated with 0.08, 0.313, 1.25, 5 uM of doxorubicin, Sp1 levels did not change. p53 was only induced in cells treated with 1.25 and 5 uM doxorubicin (Fig. 3d). Knockdown of p53 reduced p21 levels in HCT116 WT and PTTG1^−/−^ cells, and the number of cells exhibiting senescence were reduced from 58% to 27% with TSA treatment as assessed by β–Gal staining (Fig. 3b). Knockdown of Sp1 had a similar effect, with cell senescence observed in 25% of cells. Sp1 and p53 protein levels did not differ after treatment with 0.005–0.02 uM doxorubicin or TSA (Fig. 3d), suggesting that p21 up-regulation is not associated with Sp1 or p53 level changes. We then evaluated the Sp1 or p53 binding on p21 promoter. p21 promoter contains several Sp1 and p53 binding sites (Fig. 4a). We designed 2 primer pairs spanning −2500∼+50 bp of the p21 promoter (Fig. 4a), with primer pair 1 for p53 and primer pair 2 for Sp1 binding evaluation. The difference in p53 binding between doxorubicin-treated WT and PTTG1^−/−^ cells was small (1.7 vs 2.4 fold, Fig. 4b). p53 binding was enhanced ∼3.4 fold in PTTG1^−/−^ cells when treated with TSA. Sp1 binding to the p21 promoter in WT but not PTTG1^−/−^ cells was enhanced ∼6.1 fold (p<0.001) by doxorubicin (Fig. 4c). TSA enhanced Sp1 binding to the p21 promoter by ∼6.7 fold (p<0.001, Fig. 4c).

As Sp1 binding motifs are essential for p21 promoter activity [38], we assessed the effects of deleting two p53 binding sites on the p21 promoter. After transfection of pGL-basic plasmid containing different p21 promoter fragments, those treated transfectants containing two intact p53 binding motifs demonstrated 2-fold higher activity in HCT116 PTTG1^−/−^ than in WT cells (Fig. 4d). Promoter constructs devoid of p53 binding motifs showed similar activity in the two cell lines, suggesting that p53 is required to regulate pGL-p21 promoter activity in both WT and PTTG1^−/−^ cells. However, when we treated WT and PTTG1^−/−^ HCT116 cells with 0.005 uM doxorubicin or TSA, neither showed enhanced pGL-p21 luciferase activity (Fig. 4d), suggesting that chromatin changes may be involved.

### Histone acetylation on the p21 promoter

**Figure 5:**
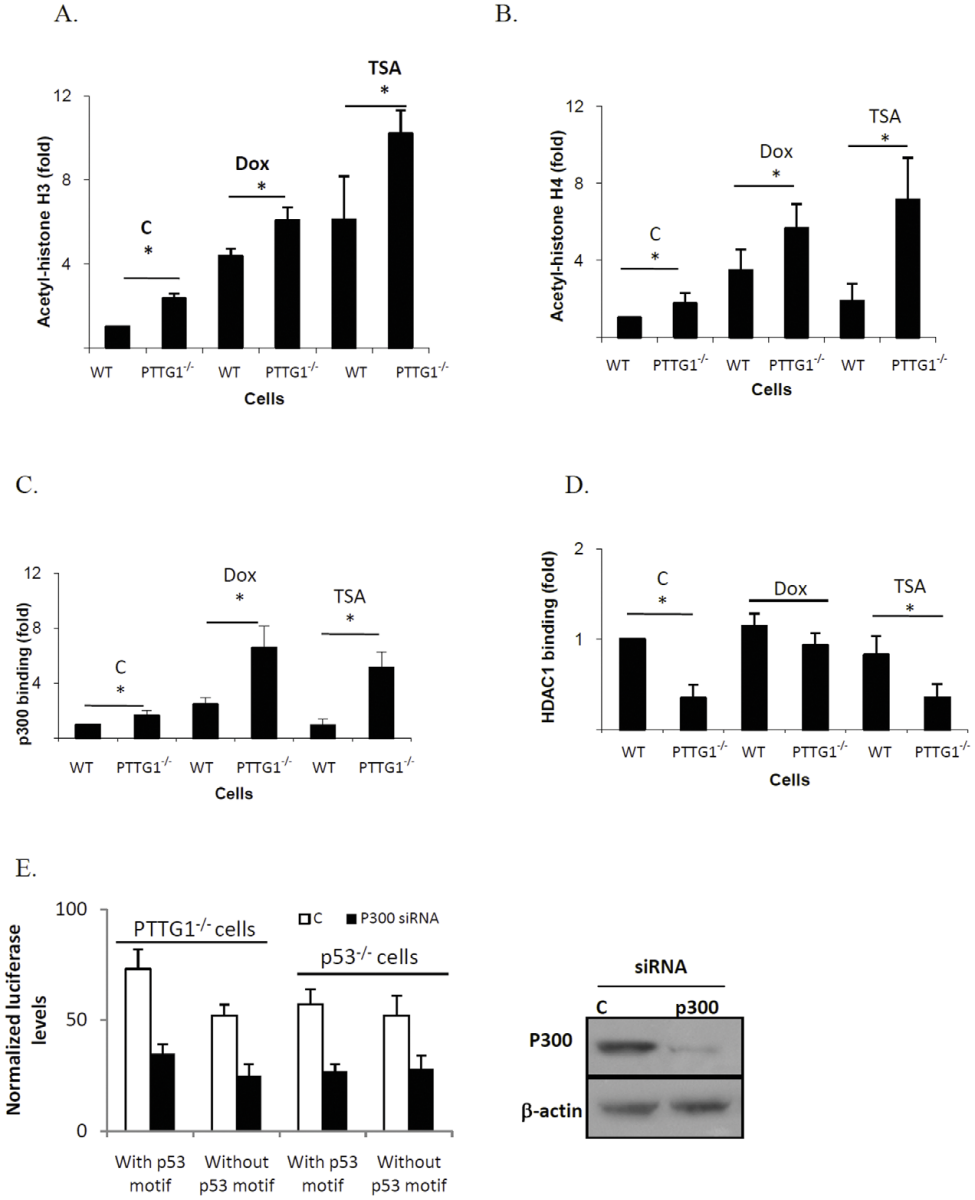
Protein levels on p21 promoter after drug treatment. a) WT and PTTG1^−/−^ cells were treated with control vehicle (C), 0.02 uM doxorubicin (Dox) or 0.005 uM TSA for 48 hours. Cells were harvested and chromatin immunoprecipitations performed using the indicated antibodies. Enriched chromatins were evaluated using qPCR and primer pair 1. Levels of p21 promoter binding were normalized to these of WT control HCT116 cells after doxorubicin or TSA treatment and are depicted as a) histone H3 acetylation; b) histone H4 acetylation; c) P300 binding; d) HDAC1 binding; e) PTTG1^−/−^ or p53^−/−^ cells were transfected with scramble (C) or p300 siRNA, and p21 promoter containing or devoid of the p53 binding site. Results are expressed as mean±SD, n = 3, *p<0.05, **p<0.001.

As histone acetylation exposes chromatin to enable protein binding to gene promoters, anti-acetylated histone H3 and anti-acetylated histone H4 were used to co-immunoprecipitate chromatin, and immunoprecipitated DNAs were evaluated using primers targeting the p21 promoter. Both primer pairs detected abundant enriched p21 promoters in HCT116 PTTG1^−/−^ cells when co-immunoprecipitated with acetylated histone H3 (Fig. 5a) or H4 antibody (Fig. 5b). Primer 1 demonstrated ∼2-fold enhanced acetylated histone H3 in HCT116 PTTG1^−/−^ cells. Doxorubicin treatment resulted in 4.3-fold increase in WT and 6.2-fold increase in HCT116 PTTG1^−/−^ cells (p<0.05), while TSA treatment resulted in 6-fold increased acetylated histone H3 in WT, and ∼10-fold increase in PTTG1^−/−^ cells (p<0.05). Primer 2 demonstrated a similar pattern (data not shown). We also used a pair of negative primers (forward: ATGGTTGCCACTGGGGATCT; reverse: TGCCAAAGCCTAGGGGAAGA) that flank genomic DNA between the GAPDH gene and the chromosome condensation-related SMC-associated protein (CNAP1) gene. As these negative primers did not detect differences between HCT116 WT and PTTG1^−/−^ cells before and after drug treatment, it is likely that enhanced histone acetylation levels observed in PTTG1^−/−^ cells are specific for these specific regions including the p21 promoter.

Histone acetylation levels are controlled by histone acetylation transferase (HAT) and histone deacetylase (HDAC) [33]. To test for global changes in HAT/HDAC activities, we measured HAT and HDAC activity in vitro, and showed no difference in WT and PTTG1^−/−^ cells (data not shown). HAT accumulation or HDAC decrease on these promoters might therefore lead to elevated histone acetylation. P300, an E1A associated protein with properties of a transcriptional adapter contains intrinsic histone acetyl-transferase activity [39] and interacts with p53 and sp1 to induce histone acetylation at corresponding regions [39], [40]. We therefore surveyed p300 binding on the p21 promoter using primer pair 1 (Fig. 5c), and show that in PTTG1^−/−^ cells p300 binding is 1.7-fold higher than in WT cells. After doxorubicin treatment, p300 binding on the p21 promoter was increased 2.5- and 6.6-fold in WT and PTTG1^−/−^ cells respectively. Cells treated with TSA demonstrated a similar enhancement of p300 binding. As HDAC-1 decreases histone acetylation levels and acts as a co-repressor with p53 and sp1 [41], enhanced histone acetylation in the p21 promoter may be due to decreased HDAC-1 binding. After doxorubicin treatment, HDAC-1 binding increased in PTTG1^−/−^ cells (from 0.35 to 0.9 fold, Fig. 5d), likely indicating a compensation for enhanced histone acetylation in the p21 promoter. HDAC-1 binding to the p21 promoter was not altered in TSA-treated HCT116 PTTG1^−/−^ cells. Similar changes were found by using primer pair 2 (data not shown).

To assess the involvement of p300 in regulating p21 promoter activity, we knocked down p300 using siRNA and evaluated p21 promoter activity through a luciferase reporter assay. As shown in Fig. 5e, knock down of p300 reduced the 3kb p21 promoter activity by ∼52% in PTTG1^−/−^ HCT116 cells. To evaluate role of p53 in p300 mediated p21 regulation, we used the p21 promoter devoid of the p53 binding site (p21-luc Δp53). P300 knock down reduced p21-luc Δp53 activity by 51% (Fig. 5e), suggesting that regulatory effects of p300 on the p21 promoter may occur independently of p53. p21-luc and p21-luc Δp53 exhibited a similar activity in HCT116 p53^−/−^ cells, further indicating that p300 regulates p21 promoter activity independent of p53.

### In vivo studies

**Figure 6:**
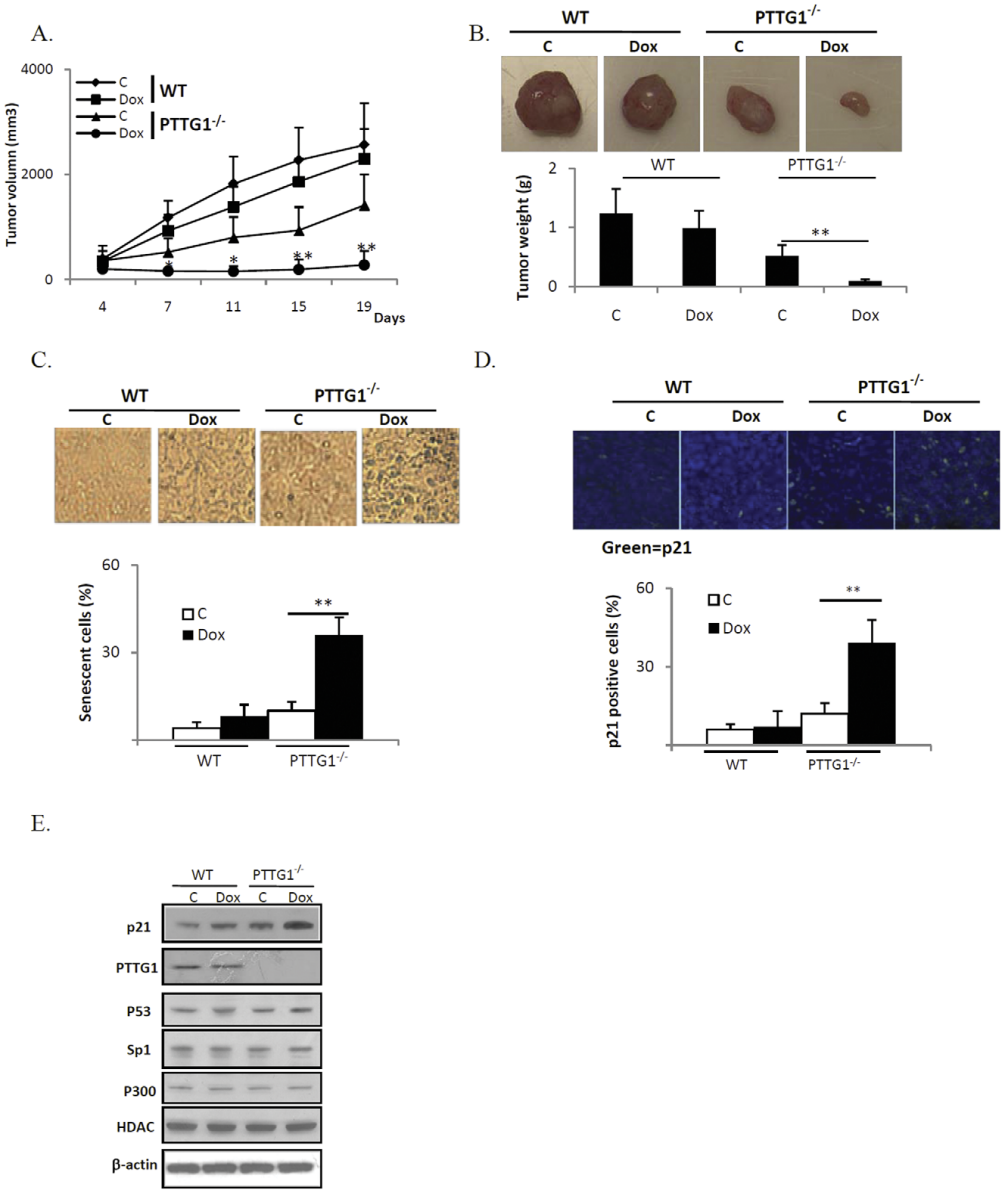
Xenografted PTTG1^−/−^ tumors are more sensitive to doxorubicin (Dox). a) HCT116 WT and PTTG1^−/−^ HCT116 cells were implanted subcutaneously into nude mice and treated with control vehicle (C) or doxorubicin. Tumor dimensions were measured twice a week; b) twenty one days after tumor cell xenografts, mice were sacrificed, tumors separated and weighed; c) harvested tumor tissues were sectioned and stained with β-gal; d) tumor tissue was stained for p21 immunoreactivity; e) harvested tumor tissues were homogenized and Western blot performed. Results are expressed as mean±SD, n = 10, *p<0.05, **p<0.001.

To test whether PTTG1-mediated senescence regulates tumor development and anti-neoplastic drug responses *in vivo*, we evaluated HCT116 WT and PTTG1^−/−^ cells using subcutaneous xenografted tumors. As shown in Fig. 6a, volumes differed in WT and PTTG1^−/−^ tumors, starting from day 7 (1237±493 vs 501±232 mm^3^, p<0.05). On day 19, the average volume of control vehicle-treated WT tumors was 2559±797 mm^3^, doxorubicin treated WT tumor was 2099±529 mm^3^ (18% inhibition compared to control vehicle -treated WT tumors), control vehicle -treated PTTG1^−/−^ tumors was 1416±585 mm^3^ and doxorubicin treated PTTG1^−/−^ tumors was 329±263 mm^3^, indicating a 77% inhibition (p<0.001) when compared to control vehicle-treated PTTG1^−/−^ tumors. Twenty one days after tumor cell implantation, mice were sacrificed, tumors harvested and weighed. PTTG1^−/−^ tumors were smaller than WT (1.32±0.39 vs 0.52±0.18 g, p<0.001) (Fig. 6b), confirming results of the tumor volume measurements. In doxorubicin treated groups, WT tumor weight was reduced to 0.99±0.29 (25% inhibition), PTTG1^−/−^ tumor weight was reduced to 0.1±0.02 g (80% inhibition, p<0.001). Body weights of control and treated mice were not different (data not shown), suggesting limited serious drug toxic effects.

Tumor tissues frozen in liquid nitrogen were sectioned and stained. As subcutaneous xenografted tumors exhibited internal necrosis (data not shown), only peripheral tumor tissue was used for subsequent analysis. In WT subcutaneous tumors, 4% tumor cells were senescent in control vehicle-treated, compared to 7% in doxorubicin-treated group. In cells derived from excised PTTG1^−/−^ subcutaneous tumors, 36% exhibited enhanced β-gal staining after doxorubicin treatment, compared to 10% in the control vehicle treated group (Fig. 6c). The number of p21 positive cells was increased in doxorubicin-treated PTTG1^−/−^ tumors (Fig. 6d). Western blotting of tumor tissue extracts showed enhanced p21 levels in PTTG1^−/−^ xenografted tumors, confirming the β-gal staining results (Fig. 6e).

## Discussion

In this study, we demonstrate that HCT116 PTTG1^−/−^ cells display enhanced sensitivity to both doxorubicin and TSA. Apoptosis, mitosis catastrophe or DNA damage were not detected, but features of senescence were observed using low doses of doxorubicin and TSA, suggesting that the PTTG1 mediated response might be due to senescence. Reintroduction of PTTG1 into HCT116 PTTG1^−/−^ cells reversed the enhanced sensitivity and features of cell senescence, suggesting that the PTTG1 effect is specific. We also evaluated PTTG1 functions in SW620 cells. Knock down PTTG1 enhanced drug-induced senescence, confirming that PTTG1 functions to suppress drug-induced senescence.

Senescence is a tumor suppressive program. It restrains cell division, and is induced by age-linked telomere shortening, oncogene activation, DNA damage, oxidative stress or chemotherapy [42], [43]. Several lines of evidence indicate that senescence contributes to cancer cell response to treatment. Senescence is important for in situ radio-sensitization of human prostate cancer cells [44], and mice bearing tumors developing drug-induced senescence have enhanced prognosis following chemotherapy as compared to those harboring tumors with defective senescence mechanisms [45]. The clinical doses of doxorubicin (0.13 to 1.5 nmol/g) [46] were found to cause senescence *in vitro* [32], suggesting a role for senescence in regulating doxorubicin efficacy.

In addition to senescence, other mechanisms have been proposed for PTTG1 regulation of drug sensitivity. PTTG1 interacts with p53 to inhibit cancer cell apoptosis [16], and with the Ku heterodimer to inhibit DNA damage repair [47]. Cells devoid of PTTG1 demonstrated impaired non-homologous DNA end-joining and therefore may regulate cell responses to DNA damage-inducing agents [48]. However, we did not detect DNA damage caused by doxorubicin or TSA when treated with indicated doses, which are in consistence with previously reported [35].

As previous research in our laboratory showed p21 plays a key role in regulating senescence [20], [27], [49], we focused our research on investigating roles of PTTG1-p21 in drug-induced senescence. Low doxorubicin concentrations (0.005–0.02 uM) induced histone acetylation and promoted p21 expression, likely responsible for enhanced cell senescence. In HCT116 PTTG1^−/−^ cells, knock down of p21 levels reduced the number of senescent cells, suggesting that p21 is critical for the enhanced senescence observed in HCT116 PTTG1^−/−^ cancer cells. The specificity of this pathway was confirmed using TSA, which also induces p21 and histone acetylation levels in treated cells. However, knock down of p21 does not completely rescue features of enhanced senescence, which could be either due to an incomplete knock down or activation of other senescence pathways. The latter is more likely because HCT116 colon cancer cells devoid of p21 are senescent when treated with anti-cancer drugs [50].

Interestingly, although p21 levels are higher in HCT116 PTTG1^−/−^ cells when treated with low dose doxorubicin and TSA, the p21 promoter did not show enhanced activity under the same treatments, suggesting that the p21 promoter luciferase assay did not fully recapitulate the p21 transcription process. Our results suggest higher levels of p53, Sp1, p300, acetylated histone H3 and H4, and lower levels of HDAC1 on the p21 promoter in PTTG1^−/−^ cells. Both p53 and Sp1 recruit HDAC1 and p300 to the promoter region [39]. PTTG1 binds p53 and Sp1 and modulates their functions [9], [16]. The results here showed more abundant p53, Sp1 and p300 on the p21 promoter in HCT116 PTTG1^−/−^ cells. Whether PTTG1 forms a transcription complex with these factors on the p21 promoter requires further investigation.

PTTG1 promotes tumor angiogenesis [24], but mechanisms underlying PTTG1-mediated angiogenesis, cell death and drug responses *in vivo* are unclear. As we observed enhanced drug-induced senescence both *in vitro* and *in vivo*, we reasoned that senescence may be a major cell arrest pathway involved. Although clinical doses of doxorubicin cause senescence *in vitro* [32], [46], the true effect of low doses of doxorubicin requires further *in vivo* study. In conclusion, these results demonstrate that PTTG1 regulates drug-induced senescence, providing new insights for the role of PTTG1 in determining cancer cell responses to anti-neoplastic treatments.

## Supporting Information

Figure S1 HCT116 PTTG1^−/−^ cells are more sensitive to doxorubicin (Dox). a) HCT116 WT and PTTG1^−/−^ cells expressing an empty vector (WT+EV and PTTG1^−/−^ +EV), and PTTG1 re-introduced PTTG1^−/−^ (PTTG1^−/−^ +PTTG1) cells were plated in 96-well plates and DNA synthesis assessed at different time points (time 0 represents 0 hours after cell plating); b) HCT116 WT and PTTG1^−/−^ cells expressing an empty vector (WT+EV and PTTG1^−/−^ +EV), and PTTG1 re-introduced PTTG1^−/−^ (PTTG1^−/−^ +PTTG1) cells were plated in 96-well plates for 24 hours and treated with control vehicle (C) or different doxorubicin doses for another 48 hours. DNA synthesis was assessed by measuring BrdU incorporation.
(TIF)

Figure S2 HCT116 cells were transfected with pEGFP-N1 plasmids and positive cells counted. Transfection efficiency was calculated by using the formula: number of EGFP-positive cells/Total cell number*100%.
(TIF)
