# Supplementary figures and images for: PTTG1 Attenuates Drug-Induced Cellular Senescence
Source: PLoS One. 2011 Aug 17;6(8):e23754. doi: 10.1371/journal.pone.0023754 (PMC3157437; doi:10.1371/journal.pone.0023754)

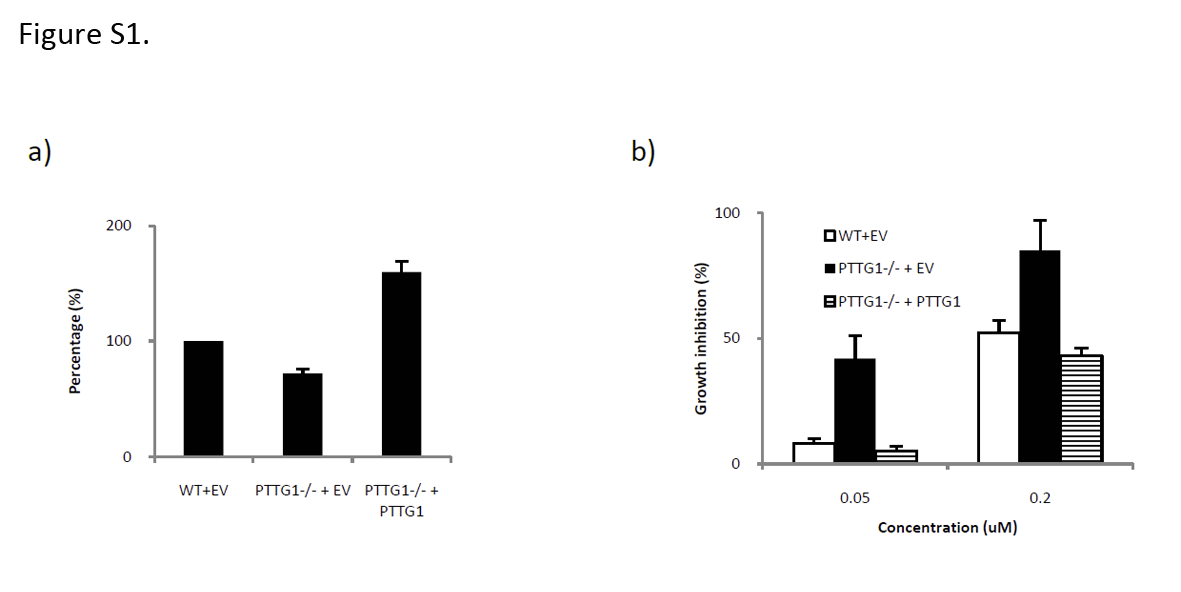

Supplement: Figure S1 — HCT116 PTTG1−/− cells are more sensitive to doxorubicin (Dox). a) HCT116 WT and PTTG1−/− cells expressing an empty vector (WT+EV and PTTG1−/− +EV), and PTTG1 re-introduced PTTG1−/− (PTTG1−/− +PTTG1) cells were plated in 96-well plates and DNA synthesis assessed at different time points (time 0 represents 0 hours after cell plating); b) HCT116 WT and PTTG1−/− cells expressing an empty vector (WT+EV and PTTG1−/− +EV), and PTTG1 re-introduced PTTG1−/− (PTTG1−/− +PTTG1) cells were plated in 96-well plates for 24 hours and treated with control vehicle (C) or different doxorubicin doses for another 48 hours. DNA synthesis was assessed by measuring BrdU incorporation. (TIF) [file pone.0023754.s001.tif]

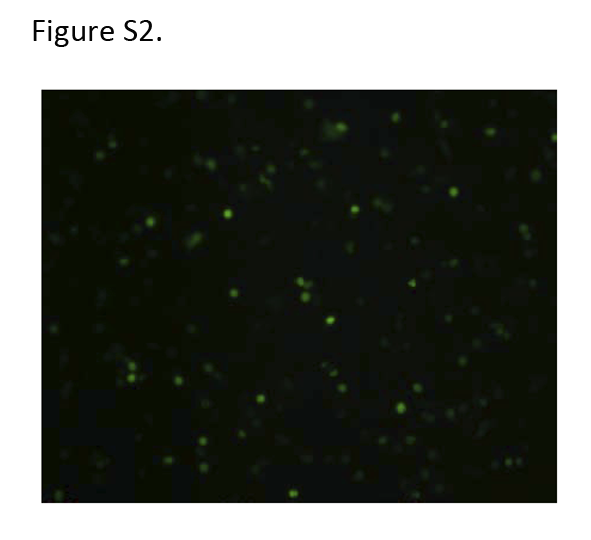

Supplement: Figure S2 — HCT116 cells were transfected with pEGFP-N1 plasmids and positive cells counted. Transfection efficiency was calculated by using the formula: number of EGFP-positive cells/Total cell number*100%. (TIF) [file pone.0023754.s002.tif]
